# Study of the effect of Lactobacillus *crispatus* FSCDJY67L3 on *Helicobacter Pylori* eradication: a double-blind randomized controlled clinical trial

**DOI:** 10.3389/fimmu.2023.1265995

**Published:** 2023-10-26

**Authors:** Qing Hong, Jidong Wang, Huayue Zhang, Xiaoming Liu, Zhenmin Liu

**Affiliations:** ^1^ State Key Laboratory of Dairy Biotechnology, Shanghai Engineering Research Center of Dairy Biotechnology, Dairy Research Institute, Bright Dairy & Food Co., Ltd., Shanghai, China; ^2^ State Key Laboratory of Food Science and Technology, School of Food Science and Technology, Jiangnan University, Wuxi, China

**Keywords:** Lactobacillus crispatus, Helicobacter pylori, eradication, co-aggregation, load

## Abstract

*Helicobacter pylori* (*H. pylori*) is a gram-negative bacterium exhibiting high pathogenicity. Traditional antibiotic treatments are considered ineffective as the *H. pylori* resistance has increased. Recently, a quadruple therapy strategy of probiotics and antibiotics to eliminate *H. pylori* was proposed. Probiotics play a therapeutic role as supplements in this process. The present research screened a probiotic strain (*Lactobacillus crispatus* FSCDJY67L3) that co-aggregates strongly with *H. pylori*. *L. crispatus* FSCDJY67L3 was demonstrated to significantly reduce *H. pylori* load (^14^C breath test) in clinical trials with *H. pylori-*positive patients. The Gastrointestinal Symptom Rating Scale (GSRS) score decreased, indicating improvement in the gastrointestinal discomfort of patients. Furthermore, *L. crispatus* FSCDJY67L3 showed no change in the structure of the intestinal flora of patients. Routine blood indices and blood biochemical indices related to liver and kidney function were also not affected in the patients. Therefore, *L. crispatus* FSCDJY67L3 may be used clinically as a supplement for the treatment of *H. pylori*.

**Clinical Trial Registration:**

https://www.chictr.org.cn/, Chinese Clinical Trial Registry (ChiCTR2100053710).

## 1 Introduction

*H. pylori*, a gram-negative bacterium, is typically parasitic in human gastric mucosal tissue (1). It is claimed that more than 50% of the population is infected with *H. pylori* at the global level (2). In particular, infection rates are significantly higher in developing countries (70-90%) than in developed countries (25-50%) (3). *H. pylori* is highly pathogenic and is the main cause of active gastritis, gastrointestinal ulcers, and gastric lymphoma (4). Furthermore, it is identified as a group 1 carcinogen by the World Health Organization (WHO) (5). Therefore, the clearance of *H. pylori* is essential for the prevention and treatment of these diseases. In the 1990s, triple therapy with antibiotics was the standard of treatment in most parts of the world. However, with the increase in clarithromycin resistance, the success rate of triple therapy has rapidly decreased (6, 7). Furthermore, triple therapy is expensive and causes serious side effects (8). Therefore, it is necessary to explore novel alternative treatment options which address the *H. pylori* epidemic.

Numerous studies have focused on the prevention and treatment of *H. pylori* by probiotics in the past few years. Recently, *H. pylori* treatment guidelines issued by the European Helicobacter and Microbiota Study Group, an authoritative group on the subject, suggest that certain specific probiotics are worth considering as an add-on to protect vulnerable patients who are poorly tolerant to antibiotics (1, 5). Among these probiotics, *Lactobacillus* is considered to be potentially effective due to the capability of these bacteria to inhibit *H. pylori*, which has been demonstrated by numerous *in vivo*/*in vitro* experiments (9). Most significantly, the inhibition mechanism of *H. pylori* by *Lactobacillus* has been elucidated. In brief, the mechanism can be summarized by the following points: (i) the intake of *Lactobacillus* stabilizes the intestinal mucosal barrier by producing antimicrobial substances that bind to *H. pylori* adhesion receptors (e.g., *Lactiplantibacillus pentosus* SLC13) (10); (ii) *Lactobacillus* may prevent *H. pylori* colonization of the gastric mucosa by inhibiting its adhesion to epithelial cells (e.g., *Lactobacillus gasseri* Kx110A1) (11); and (iii) the mucosal barrier is strengthened by *Lactobacillus* through stimulation of the local lgA response, which decreases the inflammatory response associated with *H. pylori* infection (e.g., *Lactobacillus Rhamnosus* JB3) (12, 13). Based on this, *Lactobacillus* could potentially be an effective strategy for the eradication of *H. pylori*.

The colonization of *H. pylori* with gastric epithelial cells means that it is possible to escape removal by host clearance mechanisms (14). Hence, numerous researchers were focused on preventing the colonization of *H. pylori*. The contact of *Lactobacillus* with *H. pylori* to form a copolymer may be an effective method to stop the colonization of *H. pylori*. According to Holz et al. (15), colonization is reduced by *Lactobacillus* recognizing *H. pylori* and attaching to its surface, causing it to be excreted as a co-aggregate. Therefore, the screening of a *lactobacillus* that could effectively form a co-aggregation with *H. pylori* should be the focus of research. In this paper, we found that the *L. crispatus* FSCDJY67L3 strain is able to form a copolymer with *H. pylori*. The effect of *L. crispatus* FSCDJY67L3 on *H. pylori*-positive patients via clinical trials was evaluated, which aimed to clarify whether *L. crispatus* FSCDJY67L3 has the potential to prevent and treat *H. pylori*.

## 2 Materials and methods

### 2.1 Strains and cultivation

The strain was isolated from the feces of a 90-year-old woman in Du Jiang Yan City, Sichuan. 16 S-rDNA sequence analysis allows taxonomic identification of *Lactobacillus* strains to the species level (sequencing and taxonomic classification performed by Pasono Bio, Shanghai). The sequences were aligned in GenBank and the results showed that the strains were all *Lactobacillus crispatus*, which was named *L.crispatus* FSCDJY67L3. The strain was deposited on 17 October 2022 at the China General Microbial Strain Deposit and Management Center (CGMCCNo.25925). In this research paper, *L. crispatus* FSCDJY67L3 and *Lactobacillus* strains (strains from Jiangnan University, Wuxi, China) were grown in MRS medium at 37°C. Moreover, *H. pylori* SS1 (from the National Centre for Type Culture Collection) was grown in Brain-Heart Infusion broth with 5% fetal bovine serum at 37°C in a micro-oxygenated atmosphere (85% N_2_, 10% CO_2_, 5% O_2_). The *Lactobacillus* strains and *H. pylori* bacteria were harvested by centrifugation at 8000 g for 10 min at 4 °C. They were subsequently washed twice with PBS and suspended in PBS and pH 4 artificial gastric juice (containing 0.9% NaCl and 0.3% pepsinogen), respectively, to achieve a concentration of 1×10^9^ CFU/mL for each sample.

### 2.2 Strains co-aggregation capacity

The co-aggregation ability analysis was performed according to Collado et al. (16). Briefly, lactic acid bacteria suspension (2 mL) and *H. pylori* SS1 suspension (2 mL) were mixed as well as vortexed for 10 s to determine co-aggregation capacity. The mixture was incubated at 37°C for 2 h, and the OD600 value of the bacterial solution was measured. The co-aggregation ability was calculated by the equation as follows:


$$Co-aggregation\;ability\;\left(\%\right)=\frac{OD_{\;Lactobacillus\;}+OD_{H.\;pylori}-OD_{mixture}}{OD_{Lactobacillus+}OD_{H.\;pylori}}\times 100\%$$

Where OD *_Lactobacillus_*, OD *_H. pylori_*, and OD _mixture_ represent, respectively, the absorbance at 600 nm of *lactobacillus*, *H. pylori*, and their mixture after incubating at 37°C for 2 h.

### 2.3 Scanning electron microscopy morphological observation of strains co-aggregation

The sample preparation method was referred to by Holz et al. (15). *L. crispatus* FSCDJY67L3 and *H. pylori* SS1 suspensions were mixed in equal volumes to induce co-aggregation. After incubating for 2 h at room temperature, bacterial co-aggregates were obtained by centrifugation. Subsequently, the co-aggregates were suspended in a glutaraldehyde fixation solution (4%) and kept in a cold room (4°C) overnight. The fixed co-aggregates were then centrifuged, and a gradient dehydration process was carried out using ethanol solutions (70%, 80%, 90%, 95%, and 100%) for 10 min each. The dehydrated co-aggregates were allowed to air-dry at room temperature to ensure complete evaporation of organic reagents. Afterward, they were encapsulated in plastic wrap to prevent sample splatter and subjected to vacuum freeze-drying for 2 days. Following freeze-drying, the samples underwent palladium sputter coating and were observed using scanning electron microscopy (SEM) (HITACHI SU8100, Tokyo, Japan) at high magnification of 5000× and 10000×.

### 2.4 Participants and ethics

From November 2021 to December 2021, the study enrolled patients aged 18 to 65 years at the Sixth People’s Hospital of Yancheng City (Jiangsu Province, China). The subjects enrolled met the following inclusion criteria: (i) men and women aged from 18 to 65 years old, half from each group; (ii) confirmed diagnosis of *H. pylori* infection after study entry (≤3 months) by 14^C^ urea breath test, rapid urease test, or histology; (iii) without symptoms of discomfort, except for *H. pylori* infection; (iv) prior anti- *H. pylori* treatment was not received; and (v) understood the details of the study and provided written informed consent. Patients were excluded if they met any of the following criteria: a history of gastrointestinal surgery (except for appendectomy), pregnancy or lactation, a history of any severe disease or condition (e.g., severe cardiovascular, endocrine, hepatic, or renal dysfunction), a mental illness that could potentially hinder collaboration, lack of self-cognitive judgment, substance abuse (alcohol or drugs), or failure to meet the study requirements. Additionally, patients who had taken antibiotics or probiotics in the month prior to inclusion were also excluded. The following criteria were used to determine patient exclusion: (a) if the subject was unwell or had special physiological changes in their body not appropriate to continue participating in the study, (b) if the subject elected to opt out or terminate their participation in the study, and (c) if the subject failed to undergo laboratory procedures, take antibiotics or other probiotic products, undergo a medical examination, and take stool or blood-related samples during the study. If the subject failed to fill in the relevant scale on time, the subject was considered lost to follow-up.

The experimental scheme was approved and implemented by the Yan Cheng People’s Hospital Research Ethics Board (approval no. of the ethics committee: ET2021085) and registered in the Chinese Clinical Trial Registry (Registration Number: ChiCTR2100053710). The Declaration of Helsinki and pertinent local laws were followed during the planning and execution of this study. All study participants signed an informed consent form.

Gastrointestinal Symptom Rating Scale (GSRS) (17): All patients attended an interview for the recall of gastrointestinal symptoms. The 15-item GSRS to assess the severity and frequency of symptoms was reported. According to the degree of score statistics (0~3 points), the score is proportional to the severity of symptoms. The following symptoms were specifically investigated: abdominal pain, heartburn, acid regurgitation, sucking sensations in the epigastrium, nausea and vomiting, borborygmus, abdominal distension, eructation, increased flatus, decreased/increased passage of stools, loose/hard stools, sense of urgency of evacuation, and feeling of incomplete evacuation.

### 2.5 Study design

In this study, a double-blind, randomized, and placebo-controlled trial was used to evaluate the eradication rate and safety of *L. crispatus* FSCDJY67L3 in patients with confirmed *H. pylori* infection. Patients randomly received 2 g probiotics (5 × 10^9^ CFU/package *L. crispatus* FSCDJY67L3) or matching placebo twice a day for one month. In general, probiotic doses ranging from 1.0 × 10^9^ to 1.0 × 10^10^ colony-forming units (CFU) per day have been shown to be well tolerated in the general population (18). Blood samples were collected prior to the experiment and after the intervention for further analysis, including blood routine and blood biochemical indexes related to liver and kidney function. In addition, we compared the changes in the intestinal flora of subjects after the *L. crispatus* FSCDJY67L3 intervention using 16S rDNA amplicon sequencing. Chao1 and Shannon indexes were used to characterize the α-diversity of the intestinal flora in subjects after the *L. crispatus* FSCDJY67L3 intervention. PCoA analysis was used to characterize the β-diversity.

### 2.6 Intestinal microbial composition

Fecal samples were collected before and after the experiment and were used to analyze changes in the composition of the gut microbiota. The resolved bacteria were extracted using the feces genome extraction kit from USA MP Company. After extraction, PCR was performed for the V3~V4 region of bacterial 16S rDNA. The PCR amplification system was as follows: 25 µL 2 × Premix Taq, 1 µL upstream primer 341F, 1 µL downstream primer 806R, 1 µL genomic DNA template, and 22 µL ddH2O. Amplification conditions were as follows: 95°C for 8 min; 95°C for 35 s, 52°C for 35 s, and 72°C for 40 s, 30 cycles; 72°C for 8 min. The gel was recovered using the ultra-thin agarose-gel purification and recovery kit from Beijing Tian Gen Biochemical Technology Co., LTD., and the purified fecal DNA was mixed according to the equal quality library samples and then sequenced by machine. QIIME1 software was used for data analysis after the microflora was disembarked.

### 2.7 Statistical analysis

SPSS22.0 software was used for data analysis, and GraphPad Prism 9 was used to make various statistical graphs. All data are expressed as mean ± standard deviation, and differences between groups were compared by one-way analysis of variance (ANOVA) with Duncan *post-hoc* analysis to correct for multiple comparisons. Differences were considered statistically significant when the *p*< 0.05.

## 3 Results

### 3.1 The analyses of co-aggregation ability

**Figure 1:**
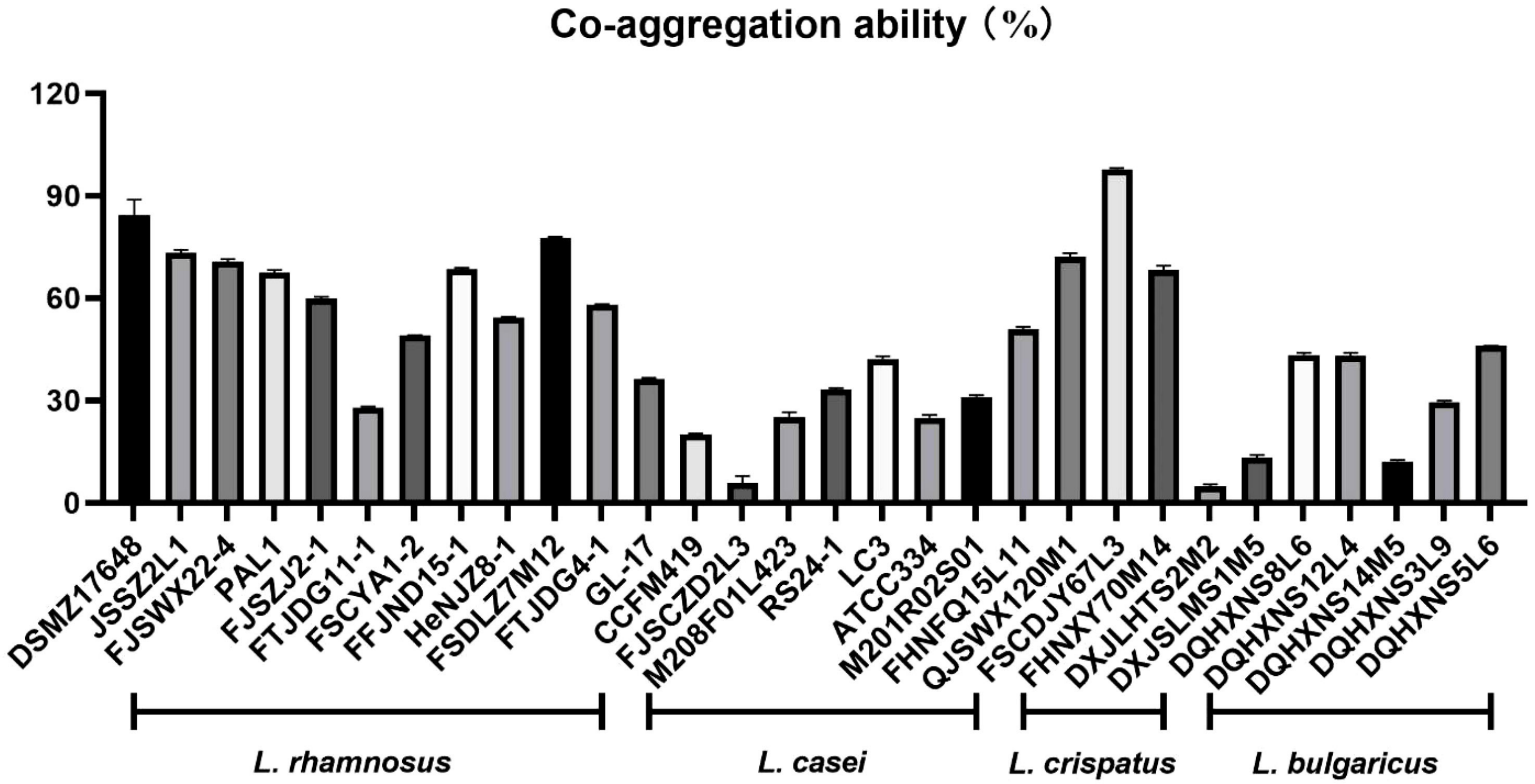
Co-aggregation ability of different strains.

In this study, *Lactobacilli* with excellent ability to co-aggregate *H. pylori* were selected from a large amount of *Lactobacillus* species (**Figure 1**). Interestingly, *L. crispatus* FSCDJY67L3 exhibited the strongest co-aggregation with *H. pylori* among all *Lactobacillus* strains. The co-aggregation rate between *L. crispatus* FSCDJY67L3 and *H. pylori* was above 97.78%.

### 3.2 SEM micro-morphological analysis of co-aggregates

**Figure 2:**
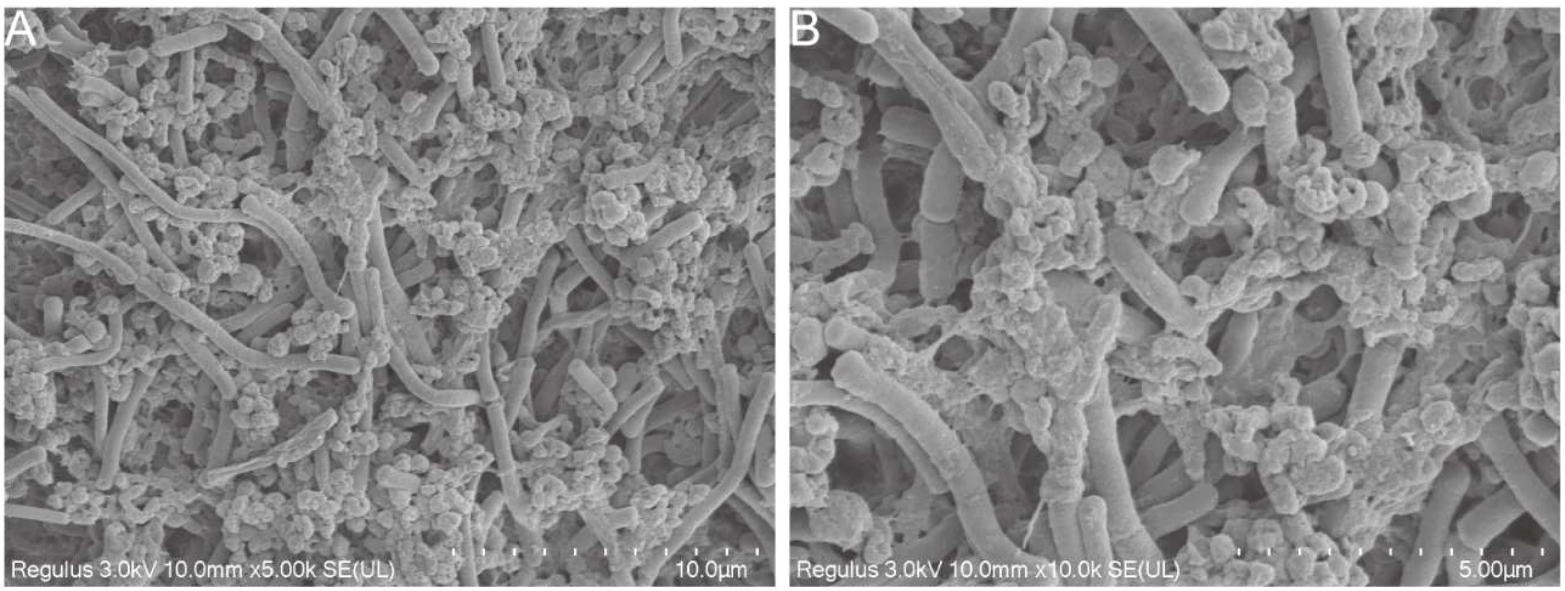
SEM morphological observation of strains co-aggregation. **(A)** 5000×magnification; **(B)** 10000×magnification.

Cold field emission scanning electron microscopy was used to observe the morphology of strain co-aggregation. In previous studies, *H. pylori* morphology was generally S-shaped or spiraled. However, it may age into a cocoon or globular shape as the environment changes. *L. crispatus* FSCDJY67L3 was long and cylindrical and co-aggregated with *H. pylori* as shown in **Figure 2**. It interacted with the surface of *H. pylori* in a strongly binding interaction. In addition, the binding site for *H. pylori* seemed to not be present on the flagellar structure of *L. crispatus* FSCDJY67L3, which is in agreement with the results reported by Holz et al. (15). Notably, *L. crispatus* FSCDJY67L3 also exhibited self-aggregation properties, suggesting that the formation of larger co-aggregates was facilitated.

### 3.3 Subjects

In the present research, patients with *H. pylori* infection (total of 44) were recruited and randomized to complete the treatment protocol with a placebo (n=20) or with *L. crispatus* FSCDJY67L3 (n=24). Unfortunately, seven patients dropped out during the process of the study, including two in the *L. crispatus* FSCDJY67L3 group and five in the placebo group. There were no patients discontinued from treatment and no loss to follow-up. The basic information on the enrolled population is shown in **Table 1**. There was no significant difference in age or sex among the groups of subjects.

**Table 1:** Background characteristics of clinical trial participants.

Group	Number of People	Sex (Male/Female)	Age (years)
Placebo	15	6/9	51.00 ± 14.25
FSCDJY67L3	22	6/16	54.91 ± 9.32

### 3.4 Effect of *L. crispatus* FSCDJY67L3 on *H. pylori* eradication rates

**Figure 3:**
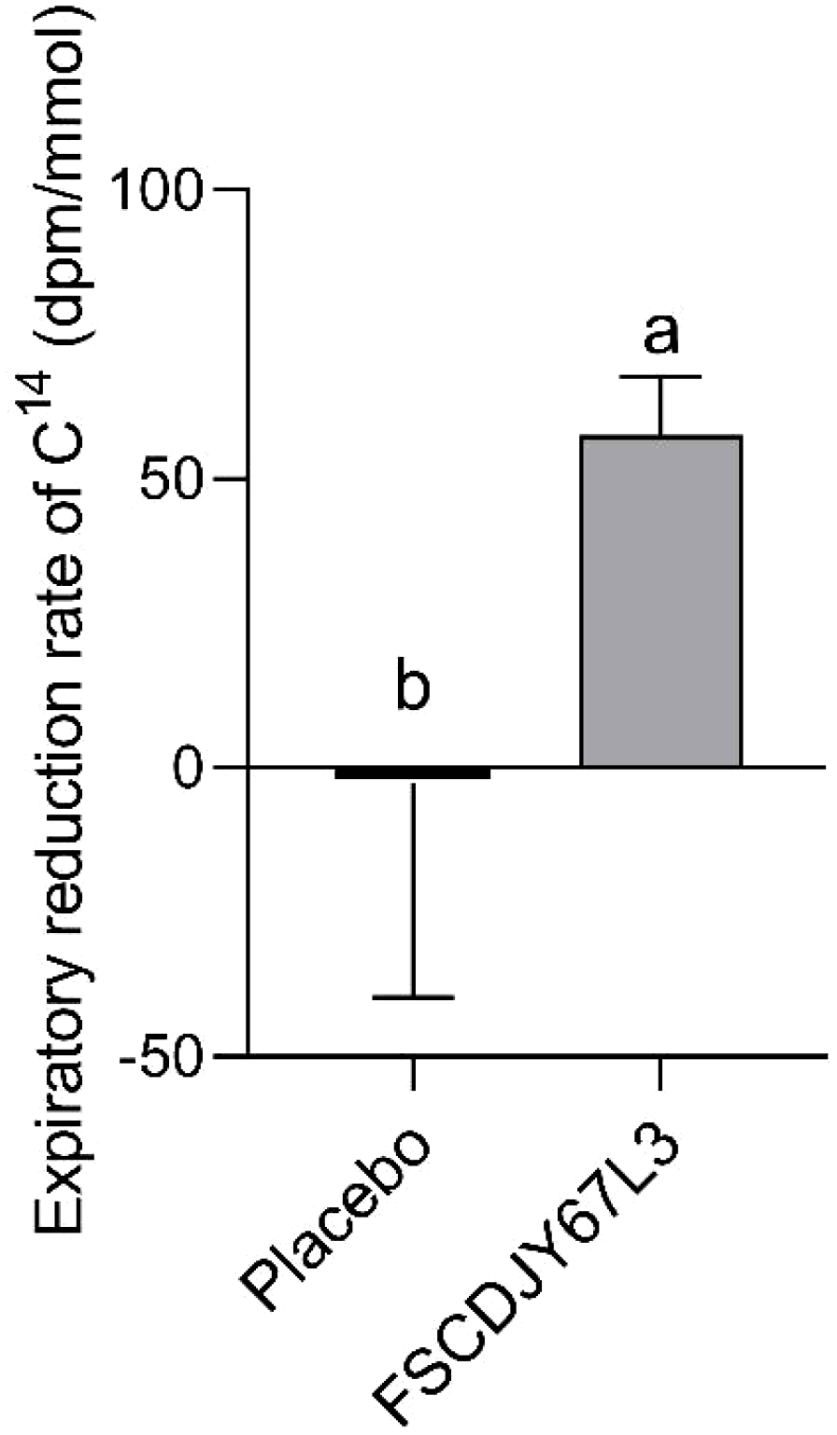
Effect of FSCDJY67L3 intervention on ^14^C expiratory value in *Helicobacter pylori*-positive patients. Different lowercase letters indicate significant differences between the two groups.

A clinical trial in patients with a family history of gastric cancer demonstrated that eradication of *H.pylori* significantly reduced the risk of gastric cancer compared to persistent infection (19). Therefore, it is crucial to decrease the load of *H. pylori* in the human organism. The expiratory value of ^14^C is a widely used clinical indicator for the diagnosis of *H. pylori* infection, which reflects the *H. pylori* load in patients. Importantly, the sensitivity and specificity of ^14^C expiratory testing usually exceed 95% (20). As shown in **Figure 3**, the reduction rate of ^14^C expiratory value in *H. pylori-*positive patients was significantly increased (67.19%) after the intervention of *L. crispatus* FSCDJY67L3. However, patients with increased ^14^C expiratory values showed a negative rate of reduction in expiratory values in the placebo group. This result indicated that *L. crispatus* FSCDJY67L3 could effectively reduce the *H. pylori* load in patients.

### 3.5 Effect of *L. crispatus* FSCDJY67L3 on gastrointestinal symptom in *H. pylori*-positive patients

**Figure 4:**
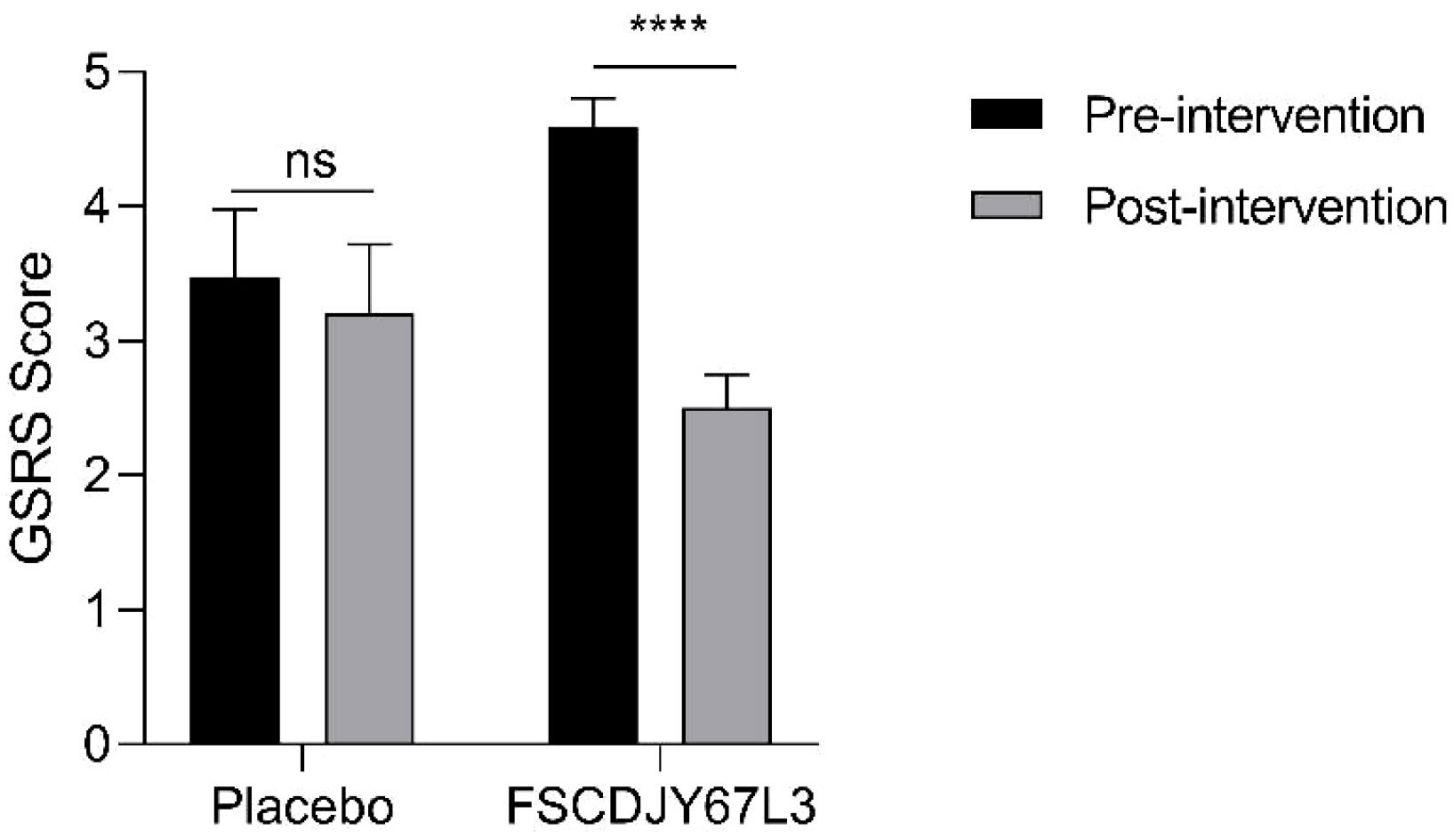
Effect of FSCDJY67L3 intervention on gastrointestinal discomfort in *Helicobacter pylori*-positive patients. ****p<0.0001. ns, no significance.

In this study, we evaluated the gastrointestinal symptoms presented by all patients, which were quantified with the GSRS score (21). There was a significant difference in gastrointestinal symptoms in *H. pylori*-positive patients before and after *L. crispatus* FSCDJY67L3 intervention. As seen in **Figure 4**, the GSRS score of the placebo group changed from 3.47 ± 2.00 to 4.59 ± 2.01 (p>0.05) after the intervention. In addition, the GSRS score of the *L. crispatus* FSCDJY67L3 group decreased from 3.2 ± 1.01 to 2.5 ± 1.19 (p<0.05), indicating that the symptoms of gastrointestinal discomfort caused by *H. pylori* were significantly improved.

### 3.6 Effect of *L. crispatus* FSCDJY67L3 on blood index in *H. pylori*-positive patients

**Figure 5:**
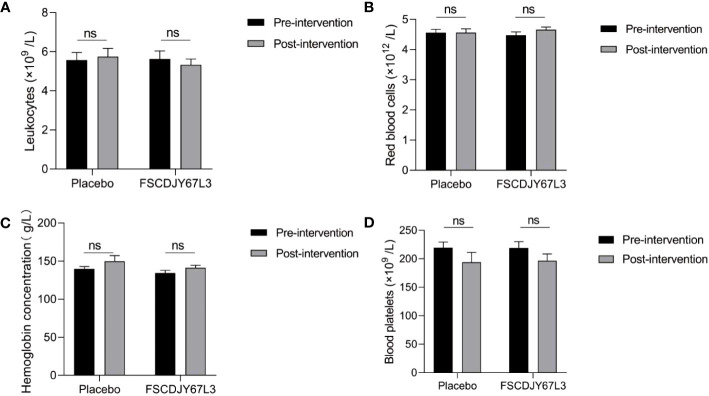
Effect of FSCDJY67L3 intervention on blood routine indexes in *Helicobacter pylori*-positive patients pre- and post-intervention. **(A)** Leukocyte count; **(B)** Red blood cells; **(C)** Hemoglobin concentration; **(D)** Blood platelets. ns, no significance.

**Figure 6:**
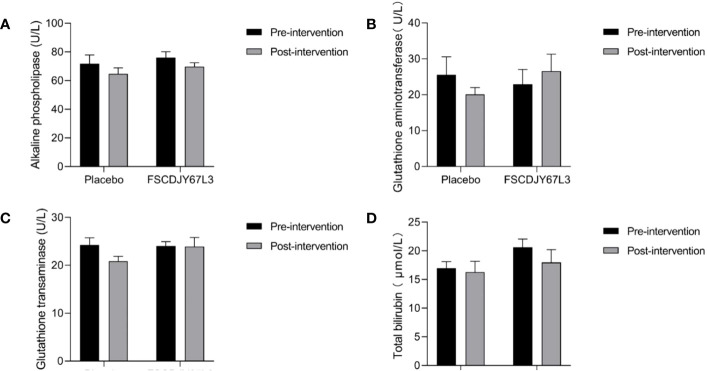
Effect of FSCDJY67L3 intervention on blood biochemical indexes refers to liver function in *Helicobacter pylori*-positive patients pre- and post-intervention. **(A)** Alkaline phospholipase content; **(B)** Glutathione aminotransferase content; **(C)** Glutathione transaminase content; **(D)** Total bilirubin.

The changes in the number of white blood cells, red blood cells, hemoglobin concentration, platelet count, the content of basic phospholipase, alanine aminotransferase, aspartate aminotransferase, and total bilirubin in patients with *H. pylori* infection in the *L. crispatus* FSCDJY67L3 and placebo groups are shown in **Figures 5**, **6**. In the placebo and *L. crispatus* FSCDJY67L3 groups, there were no significant differences between the immune mediators in the serum of the patients before and after the intervention.

**Figure 7:**
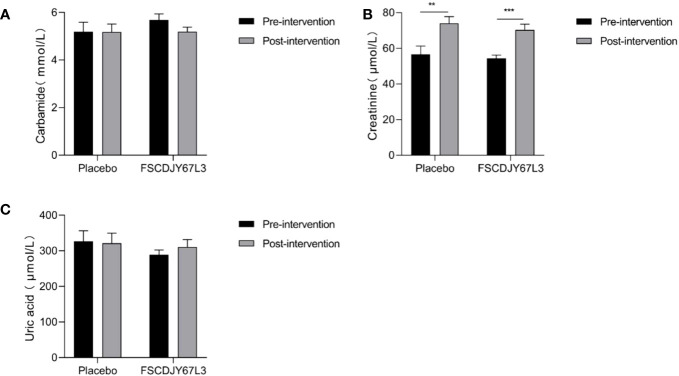
Effect of FSCDJY67L3 intervention on blood biochemical indexes refers to renal function in *Helicobacter pylori*-positive patients pre- and post-intervention. **(A)** Carbamide content; **(B)** Creatinine content; **(C)** Uric acid content. **p<0.01, ***p<0.005.

As shown in **Figure 7**, the content of urea and uric acid refers to blood biochemical indicators of renal function in *H. pylori-*positive patients and showed no significant changes before and after the placebo and *L. crispatus* FSCDJY67L3 intervention. However, in the placebo group, the creatinine content of *H. pylori*-positive patients changed from 56.62 ± 17.94 before intervention to 74.05 ± 14.51 (*p*<0.01), and that of *H. pylori*-positive patients changed from 54.46 ± 7.82 before intervention to 70.30 ± 15.07 after intervention (*p*<0.005).

### 3.7 Analysis of intestinal microbial composition in *H. pylori*-positive patients

Previous research presented the theory that changes in intestinal flora are associated with a range of gastrointestinal diseases and systemic diseases (22). In addition, Luyi et al. demonstrated that stool samples from *H. pylori*-infected individuals showed reduced abundance of Clostridium perfringens and total anaerobic bacteria as compared to *H. pylori*-negative individuals (23). Therefore, the modification of the intestinal flora could be considered as a criterion to reflect the pathological status of the organism.

**Figure 8:**
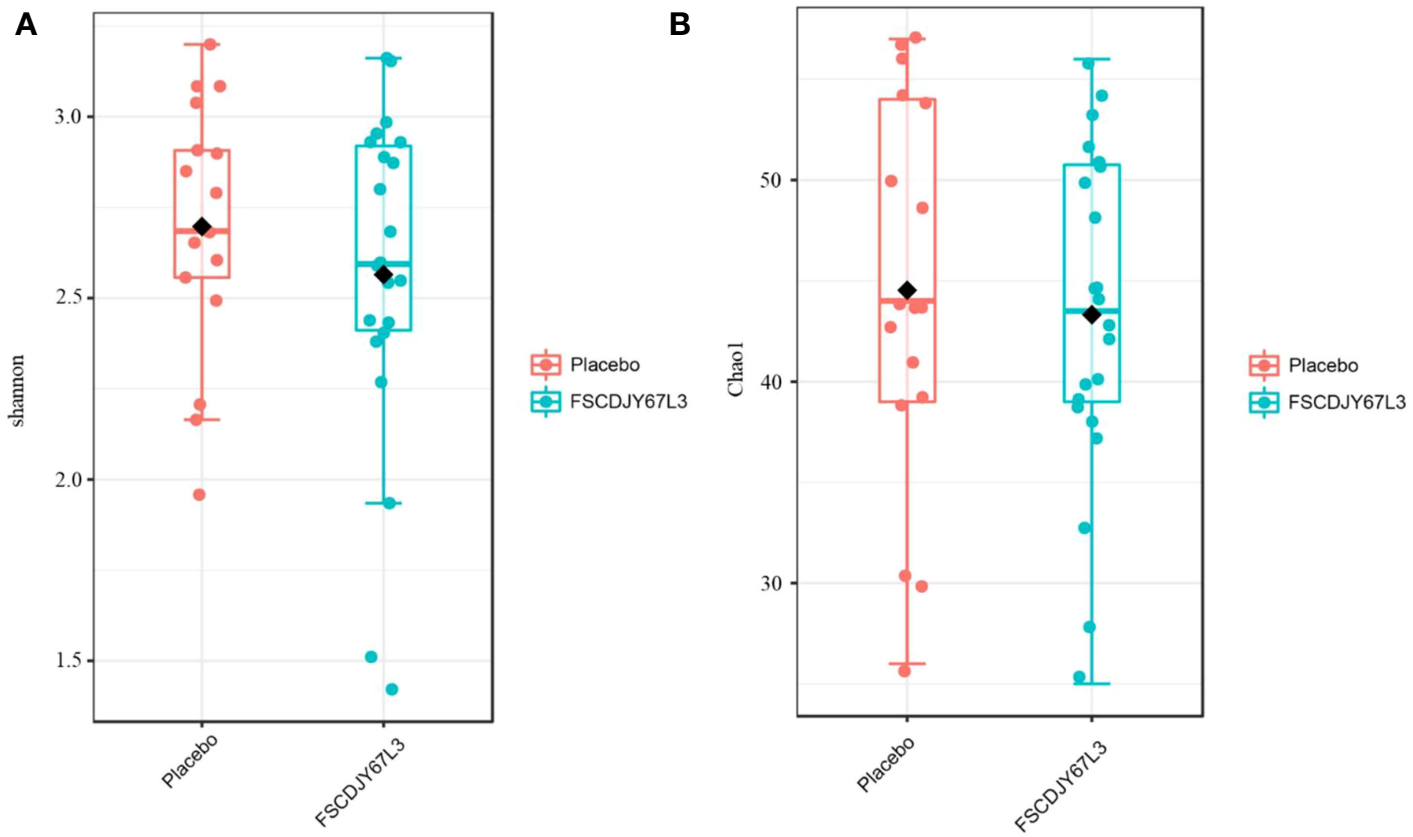
Changes of intestinal flora diversity in *Helicobacter pylori*-positive patients after intervention. **(A)** Shannon index; **(B)** Chao1 index.

**Figure 9:**
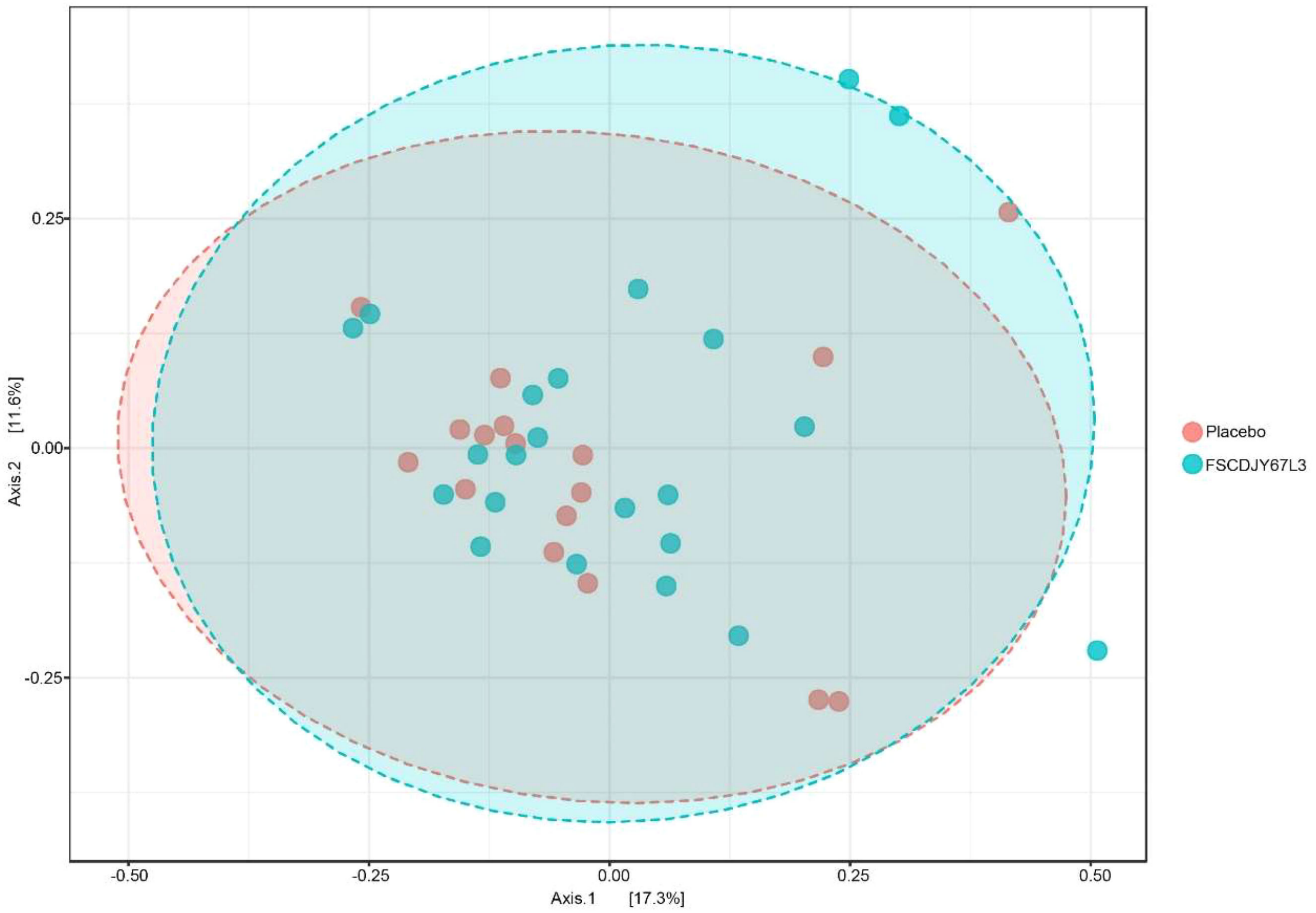
Principal coordinates analysis of intestinal flora composition in *Helicobacter pylori*-positive patients after intervention.

After intervention with the *L. crispatus* FSCDJY67L3, no significant changes in intestinal flora Chao1 and Shannon index were observed in *H. pylori*-positive infected patients (**Figure 8**), indicating that *L. crispatus* FSCDJY67L3 intervention could not alter community richness and microbial diversity in *H. pylori*-positive infected individuals. The further the distance between the different groups in the PCoA analysis indicated the greater the difference in their intestinal flora. As shown in **Figure 9**, there was no significant distance between the two groups before and after the intervention, suggesting *L. crispatus* FSCDJY67L3 could not contribute to the difference in intestinal flora in *H. pylori*-positive individuals.

## 4 Discussion

*H.pylori* is a major human pathogen, listed by the World Health Organization as one of the 20 pathogens that pose the most serious threat to human health because of its drug resistance (24). As *H. pylori* drug resistance increases, some antibiotics (e.g., amoxicillin, clarithromycin, and metronidazole) have a diminished therapeutic effect on *H. pylori*. Notably, antibiotics were considered to be able to completely eradicate *H. pylori* at an earlier time. Therefore, we need new therapeutic strategies to tackle this global problem. Recently, a therapeutic strategy was proposed, which combines probiotics and antibiotics in a quadruple therapy to completely eradicate *H. pylori*. In this process, probiotics are used as a supplement to play a therapeutic role (25, 26).

In the human intestine, *Lactobacillus* is present in high quantities and exhibits adhesion properties, allowing it to co-aggregate with pathogenic bacteria. Current research on the co-aggregation ability of *Lactobacillus* to alleviate *H. pylori* infection has primarily focused on a strain of *Lactobacillus reuteri* DSMZ17648, which was screened by German researchers (15). However, it was not yet possible to alleviate *H. pylori* infection in China by co-aggregation of *Lactobacillus* strains. This study aims to identify a *Lactobacillus* strain with superior co-aggregation ability compared to DSMZ17648 through *in vitro* screening and evaluate its effectiveness in alleviating *H. pylori* infection through clinical trials.

We characterized the co-aggregation ability of *H. pylori* and probiotic strains *in vitro* and demonstrated that FSCDJY67L3 and *H. pylori* showed the highest co-aggregation ability (27). In the DSMZ17648 clinical trial research, there was a 20.00% decrease in exhaled breath values after ingestion in subjects (28). In comparison, after the intervention by *L. crispatus* FSCDJY67L3, the breath values of patients decreased by 67.19% in the ^14^C urease breath test. This indicates that *L. crispatus* FSCDJY67L3 not only has a strong co-aggregation effect but also exhibits better clinical efficacy in alleviating *H. pylori* infection.

In this study, we monitored gastric symptoms in patients to illustrate the positive modulating effect of FSCDJY67L3. It could significantly improve the gastrointestinal symptoms in *H. pylori*-positive patients, which may be related to the reduction of *H. pylori* load in patients after ingestion of *L. crispatus* FSCDJY67L3. *Lactobacillus reuteri* DSM17938 and ATCC PTA 6475 also showed similar effects (29).

We also evaluated the safety of *L. crispatus* FSCDJY67L3 intervention by the blood routine and blood biochemical indicators of the subjects with liver and kidney function markers. There was no significant difference in the abovementioned indexes of *H. pylori*-positive patients in the placebo and *L. crispatus* FSCDJY67L3 groups except for the creatinine content. There were a number of serum markers related to the renal function that were affected in subjects after placebo or FSCDJY67L3 intervention. However, the normal reference range for creatinine in adults is 44 to 133 μmol/L, indicating that the creatinine levels of the patients were within the normal range. The variation in creatinine levels in patients may be related to their own state and physiological habits. The clinical trial of *Lb. reuteri* DSMZ17648 also showed that the intervention of *Lactobacillus* did not affect the blood routine and blood biochemical indicators as safety parameters (15), which was consistent with the results of this study. In conclusion, *L. crispatus* FSCDJY67L3 is a safe probiotic whose intervention does not affect routine blood indicators, or liver and kidney function in *H. pylori-*positive patients.

After 30 days of intervention with *L. crispatus* FSCDJY67L3, there was no significant change in the diversity of the gut microbiota in the placebo and lactobacilli intervention groups. The results are consistent with previous studies, where a short period of *Lactobacillus* intervention did not significantly affect the intestinal flora of the organism (30–32). Several long-term factors, such as genetics, environmental factors, diet, disease, and stress, determine the structure of the intestinal flora of the host (33, 34). Short-term *Lactobacillus* interventions may not significantly influence the intestinal flora of the organism. Based on this, *L. crispatus* FSCDJY67L3 is safe for humans.

The present study has a number of limitations that need to be acknowledged. We did not enroll infants and adolescents under 18 years of age in clinical trials. A study published in *The Lancet Child & Adolescent Health* suggests that the global prevalence of *H. pylori* infection in children and adolescents aged 18 years and younger is 32.3%. The rate is significantly lower than the global average value of *H. pylori* infection (35). Therefore, populations under the age of 18 years were not enrolled in our study. On the other hand, a long-term intervention clinical trial is a challenge for patient recruitment. Therefore, the clinical trial intervention period was short in this research, which led to the inability to state whether the long-term intervention would have a negative impact on the strains themselves. Moreover, only one clinical trial with a small sample size was conducted in this study. Future clinical experimental studies with large sample sizes, multi-center, and more comprehensive and rigorous designs are needed. Subsequently, synergistic effects between these strains and conventional therapeutic drugs for *H. pylori* eradication or dietary components with antagonistic effects on *H. pylori* may also be explored.

## 5 Conclusion

In this study, we found that *L. crispatus* FSCDJY67L3 exhibited a strong ability to co-aggregate with *H. pylori* in artificial gastric fluid (pH=3). Hence, it could significantly reduce *H. pylori* load and improve gastrointestinal symptoms in *H. pylori*-positive patients. On the other hand, *L. crispatus* FSCDJY67L3 showed no influence on routine blood indices and blood biochemical indices related to liver and kidney function. In addition, it also exhibited no change in the composition and diversity of the intestinal flora of the subjects before and after the intervention. Based on this, *L. crispatus* FSCDJY67L3 shows great promise in the preparation of products, such as food or pharmaceutical products, for the prevention and/or treatment of *H. pylori* infection.

## Data availability statement

The original contributions presented in the study are included in the article/supplementary material. Further inquiries can be directed to the corresponding author.

## Ethics statement

The studies involving humans were approved by The Ethics Committee of Yan cheng People’s Hospital (ET2021085). The studies were conducted in accordance with the local legislation and institutional requirements. The participants provided their written informed consent to participate in this study.

## Author contributions

QH: Data curation, Formal Analysis, Methodology, Software, Writing – original draft. JW: Formal Analysis, Writing – review & editing. HZ: Data curation, Writing – review & editing. XL: Supervision, Writing – review & editing. ZL: Conceptualization, Funding acquisition, Methodology, Writing – review & editing.
